# A *PCA3* gene-based transcriptional amplification system targeting primary prostate cancer

**DOI:** 10.18632/oncotarget.6360

**Published:** 2015-11-22

**Authors:** Bertrand Neveu, Pallavi Jain, Bernard Têtu, Lily Wu, Yves Fradet, Frédéric Pouliot

**Affiliations:** ^1^ Département de Chirurgie, Faculté de Médecine, Université Laval, Centre de Recherche du Centre Hospitalier Universitaire de Québec, Québec, Canada; ^2^ Département de Biochimie et Pathologie, Faculté de Médecine, Université Laval, Centre Hospitalier Universitaire de Québec, Québec, Canada; ^3^ Department of Molecular and Medical Pharmacology, David Geffen School of Medicine, University of California, Los Angeles, Los Angeles, California, USA; ^4^ Department of Urology, David Geffen School of Medicine, University of California, Los Angeles, Los Angeles, California, USA

**Keywords:** prostate cancer, PCA3, vector system and targeting strategies, non-invasive imaging in animal

## Abstract

Targeting specifically primary prostate cancer (PCa) cells for immune therapy, gene therapy or molecular imaging is of high importance. The *PCA3* long non-coding RNA is a unique PCa biomarker and oncogene that has been widely studied. This gene has been mainly exploited as an accurate diagnostic urine biomarker for PCa detection. In this study, the *PCA3* promoter was introduced into a new transcriptional amplification system named the 3-Step Transcriptional Amplification System (*PCA3*-3STA) and cloned into type 5 adenovirus. *PCA3*-3STA activity was highly specific for PCa cells, ranging between 98.7- and 108.0-fold higher than that for benign primary prostate epithelial or non-PCa cells, respectively. In human PCa xenografts, *PCA3*-3STA displayed robust bioluminescent signals at levels that are sufficient to translate to positron emission tomography (PET)-based reporter imaging. Remarkably, when freshly isolated benign or cancerous prostate biopsies were infected with *PCA3*-3STA, the optical signal produced from primary PCa biopsies was significantly higher than from benign prostate biopsies (4.4-fold, *p* < 0.0001). *PCA3*-3STA therefore represents a PCa-specific expression system with the potential to target, with high accuracy, primary or metastatic PCa epithelial cells for imaging, vaccines, or gene therapy.

## INTRODUCTION

Inrecent years, wide-array genomic analysis has provided a better understanding of the molecular changes occurring during prostate epithelial cell transformation and progression in aggressive cancers [1]. Exploitation of these transcriptional alterations *in vivo* is among several approaches, which can be used to better treat, prognose, or image prostate cancers (PCa) [2-4]. The prostate provides a unique anatomical location to deliver transcription expression-based nanotechnologies directly into the organ through transrectal ultrasound-guided injections, thereby avoiding systemic biodistribution and poor target delivery [5]. However, the relatively weak activity of many tumor-specific promoters has limited this approach. To circumvent this limitation, investigators have used several techniques to amplify promoter signals [3, 6, 7]. One of these approaches, the two-step transcriptional amplification (TSTA) system, has been successfully adapted for optical and PET-based molecular imaging as well as for gene therapy [7-10]. We recently demonstrated that the amplification provided by the TSTA driven by the prostate specific antigen (PSA) promoter (*PSEBC*) was strong enough to image transcriptional activity in the prostate of large immunocompetent mammals following ultrasound-guided transrectal injection and using a clinical PET imaging apparatus [5]. Despite the high translational potential of these technologies, their ability to detect primary PCa *in vivo* remains unchartered territory.

*PCA3/DD3* long non-coding RNA (lncRNA) is an oncogene overexpressed by up to 60-fold in PCa compared to benign epithelial cells [11, 12]. Its abundant expression in PCa is controlled in part through transcription and is expressed from precancerous lesions (prostatic intraepithelial neoplasia (PIN)) to metastases [13, 14]. In the clinical setting, it is used as a promising biomarker and as an adjunct to serum PSA or to magnetic resonance imaging to determine the risk of PCa prior to biopsy [15, 16]. Unfortunately, because of its weak activity, the *PCA3* promoter was not successfully exploited to target PCa cells.

In this study, we present a new transcriptional amplification system, referred to as 3STA (three-step transcriptional amplification), which boosts expression from *PCA3* promoter-directed construct up to a hundred-fold, achieving better amplification and specificity than does the current benchmark TSTA method. When driven by the *PCA3* promoter, the 3STA allows for sensitive primary PCa detection, thus significantly improves the translational potential of the *PCA3* transcription-based PCa-specific diagnosis, imaging, and therapy.

## RESULTS

### *PCA3*-3STA is a highly amplified expression system

**Figure 1:**
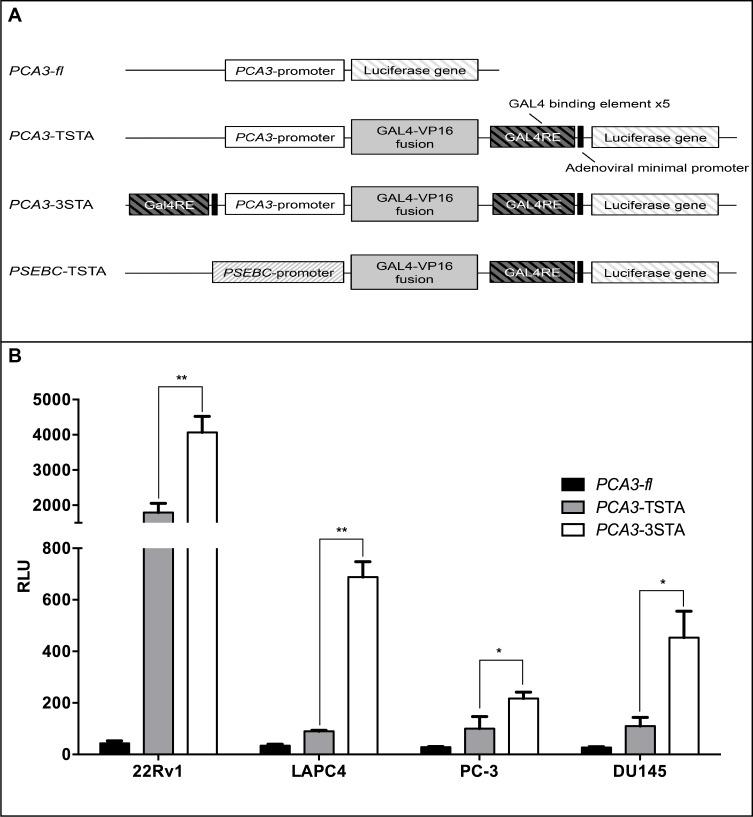
The Three-Step-Transcriptional Amplification system provides strong amplification of the *PCA3* promoter activity **A**. Scheme of non-replicative reporter adenoviruses. **B**. The amplification provided by the 3STA was significantly higher than that of the TSTA. PCa cells (22Rv1, LAPC4, PC-3, DU145) were infected with *PCA3-fl*, *PCA3*-TSTA, or *PCA3*-3STA. Cells were harvested after 72 h of infection. The relative fl activity (RLU) was normalized by protein content in each well divided by the SV40 promoter activity in each cell line (RLU = (RLU/μg protein) ÷ (RLU SV40/μg)*100). Each data represents triplicates ± SD; *p < 0.05, **p < 0.01.

Because the expression of *PCA3* lncRNA is prostate-restricted and highly amplified specifically in PCa, it is rational to exploit the specificity of its promoter in PCa diagnostic or therapeutic approaches. Unfortunately, *PCA3* promoter activity is weak and as a result has not raised much interest [17, 18]. To address this limitation, we first investigated whether its activity could be amplified once introduced within a transcriptional amplification system such as the TSTA (Figure 1A and 1B).

The TSTA system has three components: a specific promoter (e.g. *PCA3* promoter), an amplifier (GAL4VP16 fusion protein), and a reporter gene (Figure 1A). The specificity of TSTA is dictated by the promoter that drives *GAL4VP16* to initiate the transcriptional amplification of the system. Because Verhaegh et al. have shown that the minimal region (−152 to +62 bp of AF279290 sequence) of the *PCA3* promoter was more active in the PCa LNCaP cell line than other non-PCa cells, we have used this sequence as a driver of the TSTA system. As expected, when the *PCA3* proximal promoter (−152 to +62 bp) was cloned into the TSTA, its observed activity was higher than the non-amplified promoter (Figure 1B). However, the amplification provided was mild in the LAPC4, PC-3, and DU145 PCa cell lines.

To further increase the amplification provided by the TSTA, five copies of the GAL4-response elements and the adenoviral *E4* gene minimal promoter [19] were cloned upstream of the *PCA3* promoter (Figure 1A). This system, referred to as the 3-Step Transcriptional Amplification system (3STA), amplifies the *PCA3* promoter in three steps: (1) production of the GAL4VP16 fusion protein under the control of the *PCA3* promoter; (2) binding of GAL4VP16 onto the GAL4 response element (GAL4RE) upstream of the *GAL4VP16* and firefly luciferase (*fl)* genes; and (3) overexpression of the GAL4VP16 to further amplify *fl* reporter gene expression as a positive feedback loop (Figure 1A). Since the TSTA activator and amplifier cassette orientations within the viral genome have been shown to be important for signal specificity [20], we compared the activator and amplifier cassette orientations and their respective activities in four cancer cell lines (Figure S1A). As shown (Figure S1B), the tail-to-tail (TT) orientation provided the best amplification signal without changing the *PCA3*-3STA specificity for the PCa cell lines. Hence, we used the *PCA3*-3STA-TT conformation for all of our subsequent studies. As shown in Figure 1B, the fl activity of the *PCA3*-3STA is dramatically amplified. In fact, *PCA3*-3STA was shown to amplify reporter activity by 94.5- and 20.2-fold, compared to that of *PCA3*-Luc, and 2.3 and 7.6-fold compared to that of *PCA3*-TSTA in 22Rv1 and LAPC4, respectively.

### *PCA3*-3STA directs a robust expression that is specific to prostate cancer

**Figure 2:**
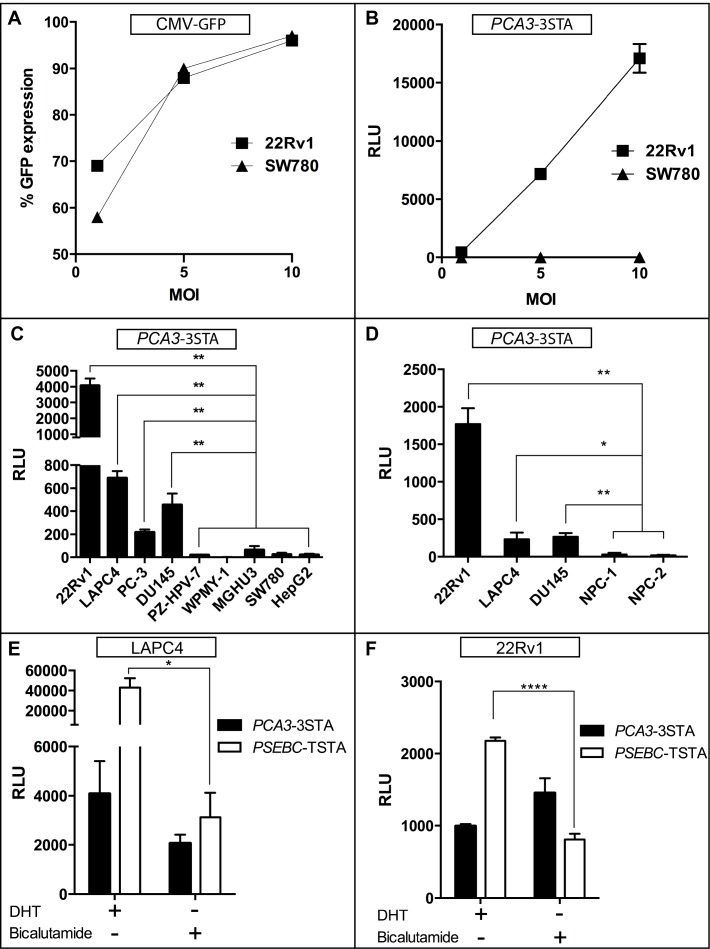
*PCA3*-3STA: A prostate- and prostate cancer-specific expression system that is not androgen-dependent **A**. *PCA3*-3STA specificity for PCa cells was not due to differences in cell line-specific infectivity. 22Rv1 (PCa) and SW780 (Bladder Ca) cells were infected with CMV-GFP adenovirus at 1, 5, and 10 MOI, and the percentage of GFP expression was found to be comparable in both cell lines. **B**. *PCA3* amplified activity in PCa specificity is not due to enhanced PCa transduction by adenovirus. 22Rv1 and SW780 cells were infected with *PCA3*-3STA at 1, 5, and 10 MOI. **C**. *PCA3*-3STA is highly active in all PCa cell lines tested. PCa (22Rv1, LAPC4, PC-3, DU145), non-PCa (SW780, HepG2), and non-cancerous prostate (PZ-HPV-7, WPMY-1) cells were infected with *PCA3*-3STA at 5 MOI. Cells were harvested after 72 h of culture. **D**. *PCA3*-3STA activity was higher in cancerous than in benign prostate epithelial cells. PCa cells and two primary prostate epithelial cell cultures were infected with *PCA3*-3STA at 5 MOI. **E**. and **F**. *PCA3*-3STA was prostate specific and not androgen dependent when compared to *PSEBC*-TSTA. Androgen receptor positive PCa cells (22Rv1 or LAPC4) were infected with either *PSEBC*-TSTA or *PCA3*-3STA at 5 MOI and treated with either DHT (10 nM) or bicalutamide (10 μM). The relative fl activity (RLU) was normalized by protein content in each well divided by the SV40 promoter activity in each cell line (RLU = (RLU/μg protein) ÷ (RLU SV40/μg)*100). Each result represents triplicates ± SD; *p < 0.05, **p < 0.01, **** *p* = < 0.0001.

Next, we assessed the PCa specificity of our *PCA3*-3STA construct by comparing its expression level in PCa cell line 22Rv1 to that in the bladder cancer cell line SW780. We first showed that adenoviral infectivity is comparable in 22Rv1 (PCa) and SW780 (bladder cancer) cells as infection with increasing multiplicity of infection (MOI) of a constitutive CMV-driven green fluorescent protein (GFP) adenovirus showed comparable GFP expression (Figure 2A). In contrast, using a *PCA3*-3STA driven *fl* adenovirus, the fl activity increased with MOI only in the PCa cells (Figure 2B). The highly amplified *PCA3*-3STA activity was restricted to PCa cell lines (22Rv1, LAPC4, PC-3 and DU145) but remained inactive in benign prostate cell lines (PZ-HPV7 and WPMY-1) and non-prostate carcinoma cell lines such as SW780 (bladder), MGHU3 (bladder) and HepG2 (hepatic) (Figure 1B and 2C). Remarkably, the 22Rv1/SW780 activity ratio (specificity ratio) increased from 2.6 for *PCA3*-Luc alone to 108.0 with *PCA3*-3STA (Figure 2C, not shown for PCA3-Luc). Furthermore, *PCA3*-3STA exhibited negligible activity in primary benign prostate epithelial cells harvested from prostate biopsies (NPC1, and NPC2, specificity ratio 22Rv1/NPC2=98.7)) which are not immortalized by viral proteins as in the case of PZ-HPV7 and WPMY-1 (Figure 2D).

Next we assessed the androgen inducibility of *PCA3*-driven amplification system, which in turn would inform us on its utility in targeting castration-resistant PCa cells (CRPC). As shown in (Figure 2E and 2F) the androgen-responsive PSA-derived *PSEBC*-TSTA activity [21] was highly induced in the presence of androgen (DHT) over that under AR blockade (with bicalutamide) in both LAPC4 and 22Rv1 (13.81- and 2.69-fold, respectively). In contrast, *PCA3*-3STA system is not androgen dependent. Upon DHT treatment of LAPC4 and 22Rv1, its activity was 1.98- and 0.68-fold that of without DHT, respectively (Figure 2E and 2F). Moreover, contrary to *PSEBC*-TSTA, *PCA3*-3STA remained active in androgen receptor (AR)-negative prostate cancer cells (PC-3 and DU145, Figure 1B and 2C) while it was silent in AR-positive breast cancer cells (CAMA-1, Supplemental Figure S2). A prerequisite of robust *in vivo* activity is the ability to maintain persistent expression in cells. Thus, we characterized the dynamics of the 3STA system in a time course experiment conducted for 96h and compared it with the TSTA system (Figure S3). *PCA3*-TSTA and *PCA3*-3STA displayed similar amplifications during 48h, after which time the *PCA3*-TSTA reached a plateau while *PCA3*-3STA activity continued to increase. This correlated with a higher level of GAL4VP16 protein at 72 and 96h for the 3STA, indicating that the enhanced amplification was due in part to the higher activation of the 3STA system by the increased GAL4VP16 protein expression (Figure S3).

To this point, our data demonstrated that the novel *PCA3*-3STA system we have generated can amplify expression via *PCA3* promoter by over 95-fold. But, importantly, this transcriptionally-based gene expression system maintained exquisite PCa-specificity that does not rely on androgens for its activity.

### *PCA3*-3STA directs a robust expression *in vivo* in PCa tumor models

**Figure 3:**
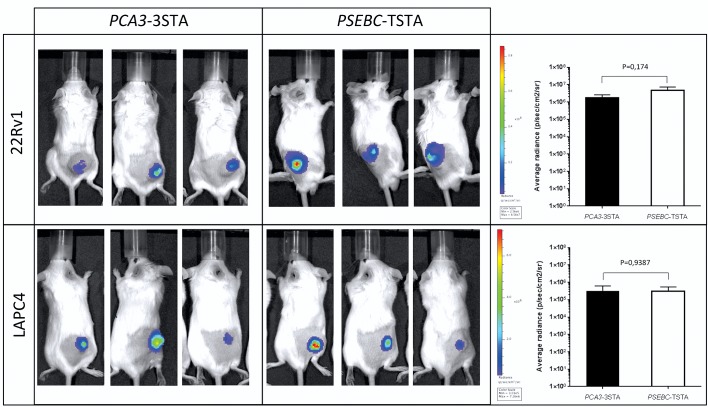
*PCA3*-3STA provided reporter expression levels *in vivo* comparable to those of the *PSEBC*-TSTA system 22Rv1 and LAPC4 PCa cell subcutaneous xenografts were generated in SCID beige mice. Following tumor growth and mice randomization for tumor volume, 10^8^ ivp of either *PCA3*-3STA or *PSEBC*-TSTA were injected intratumorally. After 96 h, reporter activity was assessed by bioluminescence. Average photon emission per tumor was measured in 22Rv1 and LAPC4 mouse xenografts (n = 3). No statistically significant difference was observed between the *PCA3*-3STA and *PSEBC*-TSTA fl activity in the two cell lines tested.

Because one of the most promising applications of the *PCA3*-3STA system is in intraprostatic imaging, we compared its activity *in vivo* with that of the *PSEBC*-TSTA system which we showed as being sensitive enough to image intraprostatic PSA promoter activity by PET in large immunocompetent mammals [5]. Figure 3 shows that following intratumoral injections of *PCA3*-3STA in 22Rv1 and LAPC4 mice xenografts, we were able to detect a strong bioluminescent signal comparable to that of the *PSEBC*-TSTA system, which suggests that *PCA3*-3STA may be sensitive enough to image intraprostatic *PCA3* transcriptional activity by PET [5].

### *PCA3*-3STA can detect primary PCa

**Figure 4:**
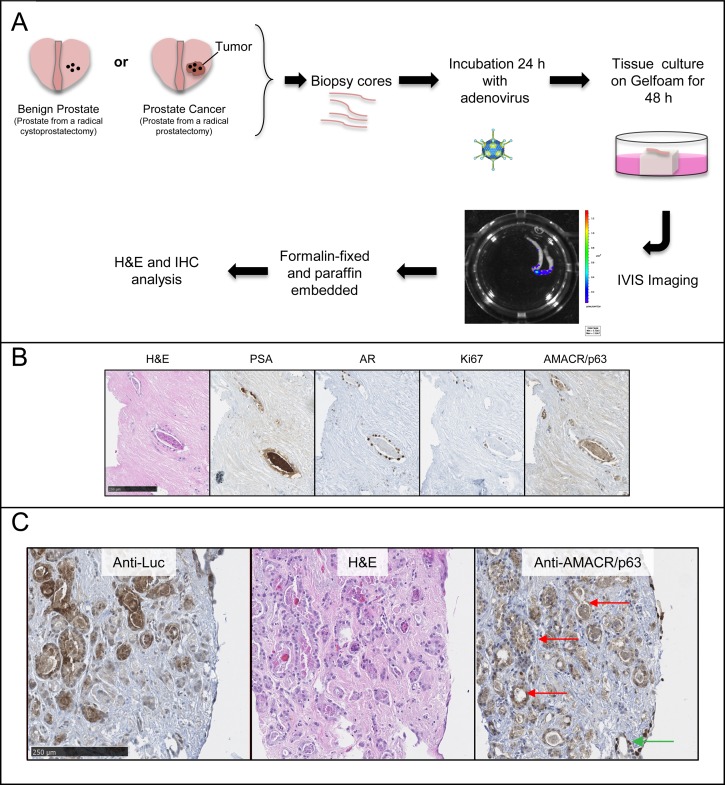
*PCA3*-3STA detected primary prostate cancer cells from radical prostatectomy specimens *ex vivo* **A**. Scheme of the technique developed. Prostate biopsy cores are harvested from radical prostatectomy (surgery performed to treat PCa) or cystoprostatectomy (surgery performed to treat bladder cancer in which the benign prostate is removed as well) specimens immediately after surgical removal. The biopsy cores are then co-cultured with *PCA3*-3STA adenovirus during 24 h after which they are cultured for 48 h on a Gelfoam sponge. Three days after infection, the luciferase activity is measured using a CCD camera and is reported as p/s/cm^2^ (Xenogen IVIS from Caliper Lifesciences). **B**. This procedure maintains PCa histological and specific biomarker expression such as prostate specific antigen (PSA), androgen receptor (AR), Ki67 or AMACR (positive in cancerous glands). **C**. Higher magnification of PCa histology and immunostainings as described in B). Note that the cytoplasmic AMACR staining is compatible with prostate cancer cells (e.g. see red arrows) while the nuclear stained cells correspond to p63 positive cells are found at biopsy periphery (see green arrow). Using this *ex-vivo* model, we show that *PCA3*-3STA-Luc adenovirus can detect primary PCa cells from radical prostatectomy specimens. Left panel: anti-firefly luciferase IHC, Middle panel: H&E staining; Right panel: anti-AMACR/p63 cocktail IHC.

**Figure 5:**
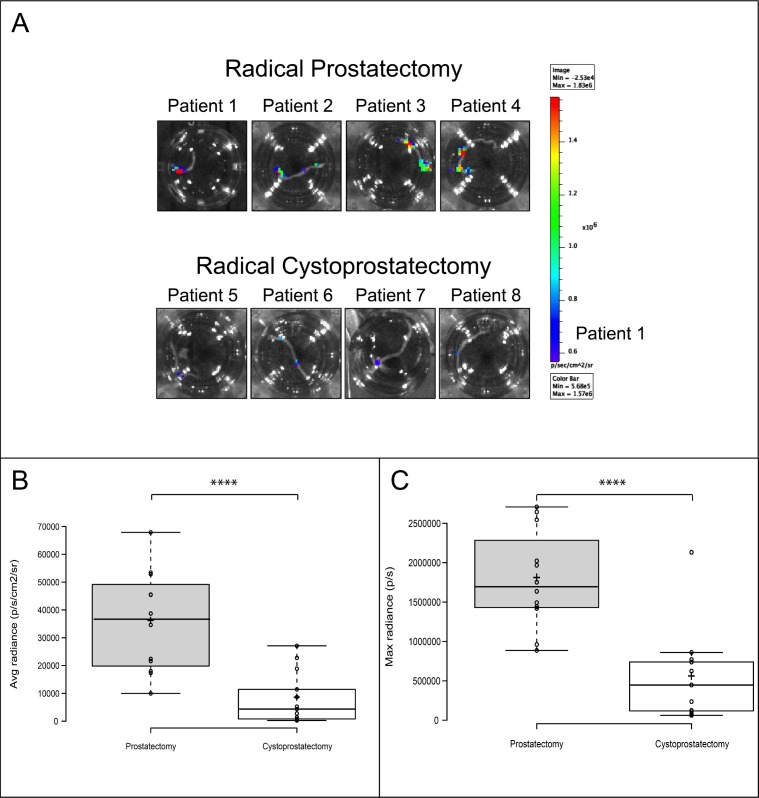
*PCA3*-3STA activity can discriminate benign from primary cancerous prostate glands Prostate biopsies were harvested from either radical prostatectomy specimens operated for prostate cancer or radical cystoprostatectomy specimens operated for bladder cancers. Each biopsy was then exposed to *PCA3*-3STA for 24 hours and then cultured for an additional 48 hours. *PCA3*-3STA activity was then quantified after luciferin addition using a BLI apparatus. **A**. Representative biopsies of radical prostatectomy or cystoprostatectomy specimens after BLI. Four patients (3 biopsy per patient) in each group were imaged and the images shown represent the biopsy with the highest radiance for each patient. **B**. and **C**. Box plots were built from the photon emission quantification ((B) Average radiance (p/s/cm^2^), (C) Max radiance per biopsy (p/s)) obtained from 12 and 13 prostate biopsies from 4 and 5 consecutive patients in the PCa and benign groups, respectively. ****p < 0.0001.

In another key step to ensure the significant clinical translational potential of this system, we tested the capacity of *PCA3*-3STA to target primary PCa cells, as most of the cell lines tested were isolated from metastatic PCa. For this purpose, biopsies were taken from cancer-bearing prostates following a radical prostatectomy or from benign prostates following a radical cystoprostatectomy (surgery performed to remove urinary bladder and prostate gland from bladder cancers patients) and the resulting samples were cultured for 72h in the presence of the *PCA3*-3STA adenovirus (Figure 4). As shown in Figure 4B and 4C, PCa cells kept PSA and AMACR expression and kept proliferating (as depicted by positive Ki67 staining) despite adenoviral infection. Moreover, the location of luciferase expression was confirmed to be located into PCa cells by IHC while stromal cells did not express the reporter gene (Figure 4B and 4C). More interestingly, a statistically significant higher fl signal was observed within the primary PCa biopsies than in the benign prostate biopsies harvested from radical cystectomy specimens (Figure 5). When the luciferase activities of *PCA3*-3STA transduced biopsies harvested from PCa patients were compared to that of non-PCa patients, the fl activity was 4.4-fold higher in the PCa biopsy-group (Figure 5A and 5B, Average radiance 35 649±17 919 p/s/cm^2^) than in the non-PCa biopsy group (Average radiance 8 072±8940 p/s/cm^2^) and the difference was statistically significant (*p* < 0.0001). Because PCa is not uniformly spread through the glands and the density of prostate epithelial cells could be responsible for the observed difference between benign and malignant tissues, we also evaluated the maximal radiance into each biopsy. Again, the radical prostatectomy group had significantly higher maximal radiance than the radical cystoprostatectomy group (Figure 5C, Max radiance=1 790 058±610 571 vs 539 638±568 550 p/s, *p* < 0.0001). As additional controls, we have infected separate biopsies from the same patients with an SV40 promoter driven *fl* adenovirus and did not observed statistical differences in the activities between the two groups (*p* = 0.27, data not shown). These results thus confirm that the *PCA3*-3STA system is indeed capable of specifically targeting primary PCa.

## DISCUSSION

In this study, we present a new transcriptional expression system, the *PCA3*-3STA, which specifically targets PCa cells in both primary PCa biopsies and PCa cell lines harvested from metastatic patients. By combining the promoter of a highly PCa-specific gene and a new transcriptional amplification system (3STA), we put forth that the *PCA3*-3STA system may be developed as a promising PCa-specific expression system not only for imaging but also for gene or immune therapy.

To obtain imageable reporter activity from a weak promoter, such as the *PCA3* promoter, we first had to construct and characterize the 3STA (Figure 1A), a derivative of the TSTA that harbours extra-Gal4 binding sites upstream of the *PCA3* promoter. This enhanced system amplifies *PCA3* promoter transcription by 95-fold (Figure 1B). Moreover, the *PCA3*-3STA is more specific for PCa cells than are the previously introduced MUC1, PSA, PEG-3 and hTERT promoter-based expression systems that can be active in many tissues or cancer subtypes [2, 22-24]. This is due in part to the observation that the 3STA not only amplified the *PCA3* promoter activity, but also maintained its specificity for prostate cancer cells. In fact, none of the many non-PCa cell lines, nor primary benign epithelial cell lines or biopsies studied (Figures 2C, 2D, S1B, S2, 5) showed significant *PCA3*-3STA activity when compared to PCa cells.

The data therefore suggest that once amplified by 3STA, the *PCA3* promoter activity displays specificity similar to that of *PCA3* lncRNA for PCa, which is known to be overexpressed from localized PCa to CRPC [11, 25, 26]. Its activity also matches that of *PCA3* RNA that does not correlate with Gleason scores or pathological stage and is consistent with a high sensitivity for PCa detection using its promoter as a driver of 3STA. The underlying mechanisms of high *PCA3* promoter specificity for cancerous prostate glands are as yet unknown. Verhaegh et al. clearly showed that the *PCA3* (−152 to +62 bp) promoter displayed the strongest activity in LNCaP cells secondary to an unidentified DNA-binding protein between the −29 and −63 nucleotides [18]. However, while the proximal *PCA3* promoter (−152 to +62 bp) did show some enhanced promoter activity in PCa cells, compared to that of benign or non-PCa cells, it was shown to be only 20 to 50% that of the weak SV40 promoter [17, 18]. Because *PCA3* lncRNA is overexpressed by 60-fold in PCa patients tissue samples when compared to benign patients tissue samples, other mechanisms than transcription seem to be involved in *PCA3* RNA overexpression in PCa [11]. Furthermore, two groups have published on an alternative *PCA3* gene transcript (*PCA3*TS4) using another transcription start site, 136 bp upstream of the start site of the promoter which we have used (*PCA3*TS5 promoter) [12, 25]. Because the promoter of the *PCA3*TS4 transcript was not included in the constructs used, this alternative promoter does not contribute to the specificity observed with the 3STA. Moreover, Salagierski et al. showed that the *PCA3*TS4 transcript expression levels accounted for only 1% of all *PCA3* gene transcripts, suggesting a low activity of the promoter upstream of this transcript (so-called *PCA3*TS4) [25].

Of interest is that the *PCA3* lncRNA (*PCA3*TS5) was androgen-induced [12, 25] whereas the *PCA3*-3STA was not (Figure 2E and 2F) which is in line with the absence of an androgen response element in the region of the *PCA3* promoter used. This is a valuable characteristic of the system, as the ability to target both androgen-sensitive and -insensitive PCa is of the utmost importance, what with the new challenges associated with CRPC metastasis detection by imaging, a prerequisite to systemic treatment. Recently, Salameh et al. have shown that the *PCA3* lncRNA was an oncogene targeting the PRUNE2 RNA, the later being a tumor suppressor gene in PCa [12]. Interestingly, *PCA3* RNA was shown to hybridize to the RNA encoding PRUNE2 gene intron 6 to induce an adenosine deaminase acting on RNA (ADAR)-dependent degradation. This important study clearly shows that *PCA3* is not only a biomarker but also a driver of PCa development and reinforce the importance to target *PCA3* overexpressing cells could definitely be exploited.

Interestingly, the PCA3-3STA system shows reporter activity comparable to that of the PSEBC-TSTA both *in vitro* (Figures 2E, 2F and S2) and *in vivo* (Figure 3). Given the extensive work performed using the PSEBC promoter in the TSTA system, it appears reasonable to assume that the gene therapy or imaging capabilities of the *PCA3*-3STA may be comparable to that of the *PSEBC*-TSTA, particularly for lymph node metastases or intraprostatic imaging by PET [5, 27]. The ability to discriminate benign from cancerous cells is particularly important in PCa, as the disease is usually low burden, multifocal, and well differentiated, all characteristics that limit accurate anatomical or metabolic imaging using classical metabolic PET tracers or magnetic resonance imaging (MRI). Moreover, as stated above, because the *PCA3*-3STA system is not designed solely for imaging, it could also be modified for gene or immunotherapy. With the recent clinical studies showing the effectiveness of the Sipuleucel-T and Prost-VAC immunotherapies in PCa, as well as the advent in clinics of checkpoint inhibitors [28], this system could be used to drive immunomodulator cytokines and/or cytotoxic genes to initiate a local or systemic immune response following intraprostatic injection. Indeed, the ability to combine transrectal intraprostatic delivery of the vector (avoiding adenoviral biodistribution problems), the PCa specificity of the *PCA3* promoter in primary PCa and the amplification provided by the 3STA make this system highly translational.

We acknowledge that our study did have its limitations. First, we did not directly demonstrate that the *PCA3*-3STA was capable of imaging *PCA3* promoter activity using a clinical PET, as previously shown with the *PSEBC* promoter. We do believe, however, that because *PCA3*-3STA showed a higher specificity and sensitivity comparable to that of the *PSEBC*-TSTA, the potential of our system for immuno- and gene therapy or imaging certainly parallels and may even exceed that of previously reported vectors [5, 17]. Moreover, given that *PCA3*-3STA was PCa-specific, it was not possible to show its activity in large immunocompetent mammals, as it was performed for *PSEBC*-TSTA, which may image AR-dependent transcriptional activity in benign prostate epithelial cells [5]. Finally, the adenovirus as a delivery method may be subject to reluctance for security and biodistribution. However, we, as well as others, have shown that the prostate allows intratumoral transrectal adenovirus delivery and is therefore a unique cancer site easy to target [29]. Also, other delivery methods such as liposomes could be used to deliver the *PCA3*-3STA.

## CONCLUSIONS

This study presents a new PCa-specific expression system, the *PCA3*-3STA, based on the *PCA3* oncogene promoter that matches the *PCA3* lncRNA expression pattern. This system may indeed be used with significant accuracy to target PCa epithelial cells for imaging, vaccines, or gene therapy applications.

## MATERIALS AND METHODS

### Plasmid construction and adenovirus production

The *PCA3* promoter (−152 to +62 bp of Genbank accession number AF279290) was PCR-amplified from human genomic DNA with the following primers: *PCA3*-Fwd, CATGTAGCAAGCTTCCATGGCATATGTGTCAAC ATAGTGTGACGGGAAG and *PCA3*-Rev, CATGTAGCAAGCTTCCACACAAATCTCCCCTCTGT [17, 18]. The *PCA3* promoter was cloned upstream of the *firefly luciferase* (*fl)* gene in pENTR-L1L2 (Life Technologies, Burlington, ON, Canada) to obtain pENTR-L1L2-*PCA3-fl*. Plasmid pENTR-L1L2-*PCA3-fl* was subcloned into pAd-pL-DEST by LR cloning (LR Clonase II Plus, Life Technologies) to obtain the adenovirus *PCA3-fl*. For the construction of the *PCA3-*TSTA and *PCA3-*3STA adenoviruses, we used the MultiSite Gateway® Pro 2.0 kit (Life Technologies). First, the pDONR221-L1L2 was used to obtain two different pENTR plasmids with multiple cloning sites, pENTR-L1R5-MCS and pENTR-L5R2-MCS, containing attL1/attR5 and attL5/attR2 sites, respectively. GAL4RE (GAL4 response elements), first component of the TSTA along with the *fl* gene, was PCR-amplified from pSh-TSTA-*fl* and cloned into pENTR-L1R5 to obtain pENTR-L1R5-GAL4RE-*fl.* GAL4VP16, second component of the TSTA, was also amplified from pSh-TSTA-*fl.* The *GAL4VP16* gene downstream of the *PCA3* promoter was cloned into pENTR-L5R2 to obtain pENTR-L5L2-*PCA3*-GAL4VP16. The pENTR-L1R5-GAL4RE-*fl* and pENTR-L5L2-*PCA3*-GAL4VP16 plasmids were subcloned into pAd-pL-DEST by LR cloning to generate *PCA3-*TSTA adenoviral plasmid. The *PCA3* promoter was replaced by the *PSEBC* promoter also amplified from pSh-TSTA-*fl* in the above-described plasmids to obtain the *PSEBC-*TSTA adenovirus. Another set of GAL4RE was cloned upstream of the *PCA3* promoter in the plasmid pENTR-L5L2-*PCA3*-GAL4VP16 to obtain pENTR-L5L2-GAL4RE-*PCA3*-GAL4VP16 which, along with pENTR-L1R5-GAL4RE-*fl*, was subcloned into pAd-pL-DEST by LR cloning to obtain the *PCA3-*3STA adenoviral plasmid. The adenoviral plasmids were then transfected into 293A cells for adenovirus production. Titers were determined using the Adeno-X™ Rapid Titer Kit (Clontech, Mountain View, CA, USA).

### Cell cultures

22Rv1 (prostate cancer cell line), CAMA-1 (breast cancer cell line), and WPMY-1 (myofibroblast stromal cell line) were cultured in RPMI media containing 10% fetal bovine serum (FBS). LAPC4 (prostate cancer cell line) and HepG2 (liver cancer cell line) were cultured in DMEM media containing 10% FBS. PC-3 and DU145 (prostate cancer cell lines), and MGHU3 and SW780 (bladder cancer cell lines) were cultured in eMEM media containing 10% FBS. PZ-HPV-7 and benign primary cell cultures NPC1 and NPC2 (harvested from prostatic biopsies) were cultured in KSFM medium (Life Technologies), as previously described [30]. This study was approved by the Institutional Review Board of the CHU de Quebec Hospital, Quebec, QC, Canada. Each patient signed an informed consent form.

### Adenoviral infection and androgen sensitivity

22Rv1 (1.6 × 10^5^/well), LAPC4 (1.6 × 10^5^/well), PC-3 (6 × 10^4^/well), DU145 (6 × 10^4^/well), PZ-HPV-7 (8 × 10^4^/well), WPMY-1 (8 × 10^4^/well), MGHU3 (8 × 10^4^/well), SW780 (8 × 10^4^/well), and HepG2 (7 × 10^4^/well) were seeded in 24-well plates. Twenty-four hours later, the *PCA3-fl*, *PCA3*-TSTA, *PCA3*-3STA, and SV40-*fl* adenovirus were added. Seventy-two hours after infection, cells were lysed and a luciferase assay was performed as described (Promega). For androgen sensitivity assessment, cells were treated with dihydrotestosterone (DHT) at 10nM or bicalutamide at 10μM (Sigma-Aldrich, St.Louis, MO, USA) in 10% charcoal-stripped FBS, 24h post-infection. Luciferase assays were performed after 48 h of treatment.

### Time course experiments and western blot analysis

22Rv1 cells (1.6 × 10^5^/well) were seeded 24h prior to infection with *PCA3-fl*, *PCA3*-TSTA and *PCA3*-3STA. Relative luciferase unit (RLU) was assayed at the indicated time points for each virus. Western blot analysis was performed on the collected lysates with 4μg of protein and the membranes were incubated with antibodies against β-actin (1:5000, Sc47778, Santa Cruz, CA, USA) or vp16 (1:5000, ab4809, Abcam, Toronto, ON, Canada). The secondary antibodies (1:5000) were conjugated with IRDye™ 680-anti-mouse and IRDye™ 800-anti-rabbit antibodies (LI-COR, Lincoln, NE, USA). Proteins were detected and the signal was quantified by means of the Odyssey Scanning System (LI-COR, Lincoln, NE, USA).

### *In vivo* experiments

Two million 22Rv1 and LAPC4 cells mixed in Matrigel (354262, Fisher Scientific, Ottawa, ON, Canada) were injected subcutaneously into the flanks of Fox Chase SCID beige mice (code 250, Charles River Canada, St-Constant, QC, Canada). Tumors were grown for 32 days. The adenoviruses (10^8^ infectious viral particles (ivp)) in phosphate buffered saline (PBS) were injected intratumorally. Ninety-six hours later, bioluminescence was quantified using a CCD camera and is reported as p/s/cm^2^ (Xenogen IVIS, Caliper Lifesciences, Woodbridge, ON, Canada). All of the animal experiments were approved by the Laval University animal care committee.

### *Ex vivo* primary PCa detection

Biopsy cores (18G, 17mm) were taken from the prostate peripheral zone following removal by robotic radical prostatectomy or radical cystoprostatectomy performed to treat PCa or bladder cancer, respectively (see Supplemental Table S1). Sextants harboring PCa at the transrectal biopsy and a nodule were targeted. The biopsies were washed with PBS and cultured for 24 hours in eMEM-10%FBS supplemented with 10nM DHT and antibiotics and the *PCA3*-3STA (10^7^ ivp) adenovirus under gentle shaking. The biopsies were then transferred on a Gelfoam soaked into fresh media for another 48h, after which time luciferin was added and the luminescence activity was imaged by means of Xenogen IVIS [31]. The biopsies were then fixed in 10% formalin and embedded in paraffin. Five-μm sections were analyzed with either H&E staining or immuno-histochemical staining (IHC). The luciferase IHC was performed with the IDetect Super Stain System HRP (IDST1007, ID Laboratories, London, ON, Canada) with anti-luciferase (1:7000, 70R12141, Fitzgerald, Acton, MA, USA) as the primary antibody and Donkey anti-Goat antibody (1:500, Ab6884, Abcam, Toronto, ON, Canada) as the secondary antibody. The IHC with the PSA, androgen receptor (AR), Ki67 or cocktail of AMACR/p63 antibodies were performed by the clinical pathology facility at the CHU de Quebec Hospital using a standard clinical protocol.

### Statistical analysis

All of the statistical analyses were conducted using the T-test with Welch's correction, with (****) indicating *p* = < 0.0001, (**) indicating *p* = < 0.01 and (*) indicating *p* = < 0.05.

## SUPPLEMENTARY MATERIAL FIGURES AND TABLE


