# Supplementary material for: A PCA3 gene-based transcriptional amplification system targeting primary prostate cancer
Source: Oncotarget. 2015 Nov 22;7(2):1300–10. doi: 10.18632/oncotarget.6360 (PMC4811461; doi:10.18632/oncotarget.6360)
Supplement: Supplementary file 1 [file oncotarget-07-1300-s001.pdf]

A *PCA3* gene-based transcriptional amplification system targeting primary prostate cancer

Supplementary Material

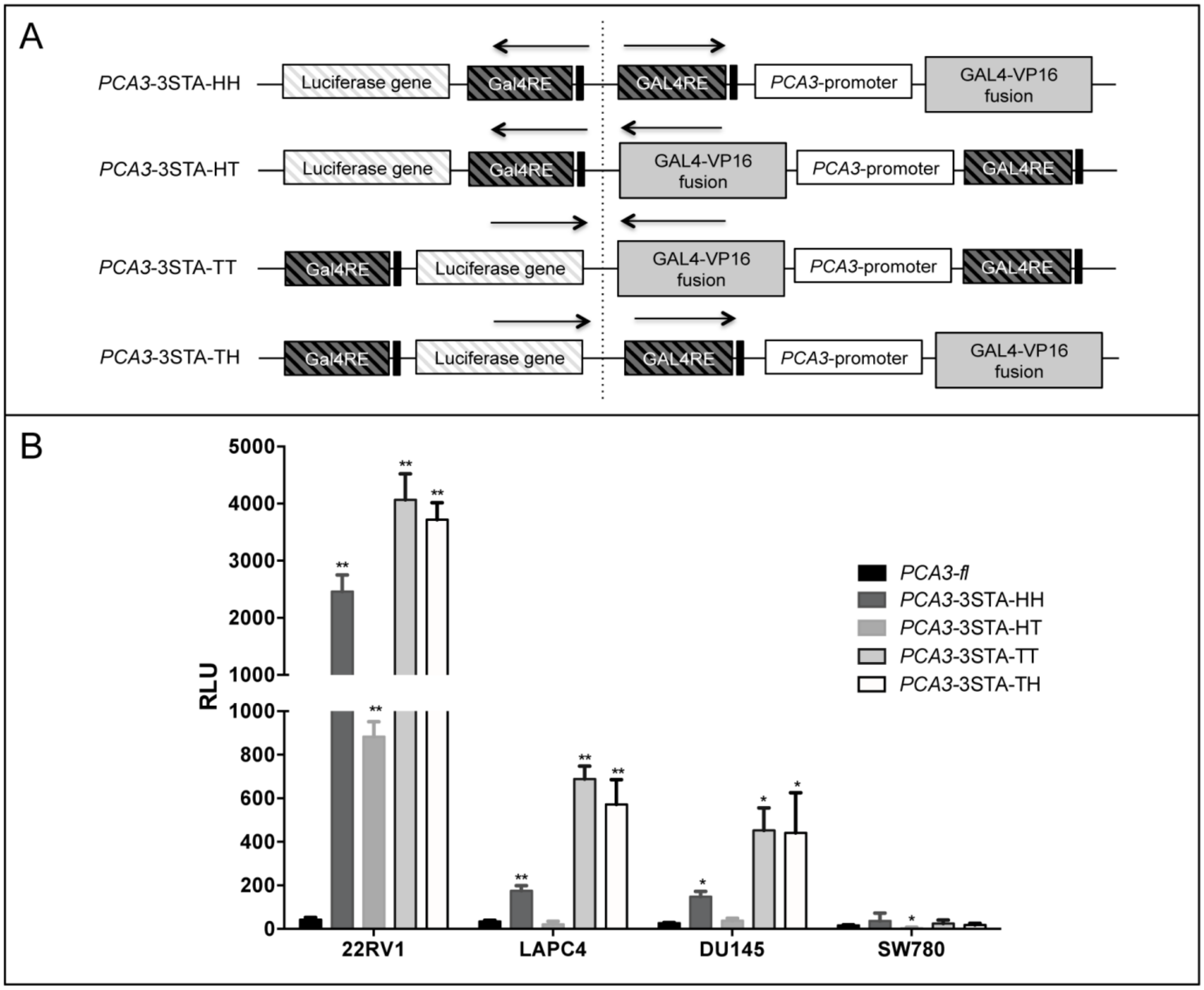

Supplementary figure 1: **Characterization of the *PCA3*-3STA conformations for prostate cancer-specific expression. (A)** Scheme of non-replicative reporter adenoviruses. **(B)** The amplification provided by the *PCA3*-3STA-TT conformation is significantly higher than *PCA3*-3STA-HH, *PCA3*-3STA-TH, or *PCA3*-3STA-HT conformations. PCa (22Rv1, LAPC4, DU145) and non-PCa (SW780) cells were infected with *PCA3-fl*, *PCA3*-3STA-HH, *PCA3*-3STA-TT, *PCA3*-3STA-TH, or *PCA3*-3STA-HT at 5 MOI**.** Cells were harvested after 72 h of infection. The relative fl activity (RLU) was normalized by protein content in each well divided by the SV40 promoter activity in each cell line (RLU= (RLU/μg protein) ÷ (RLU SV40/μg)*100).

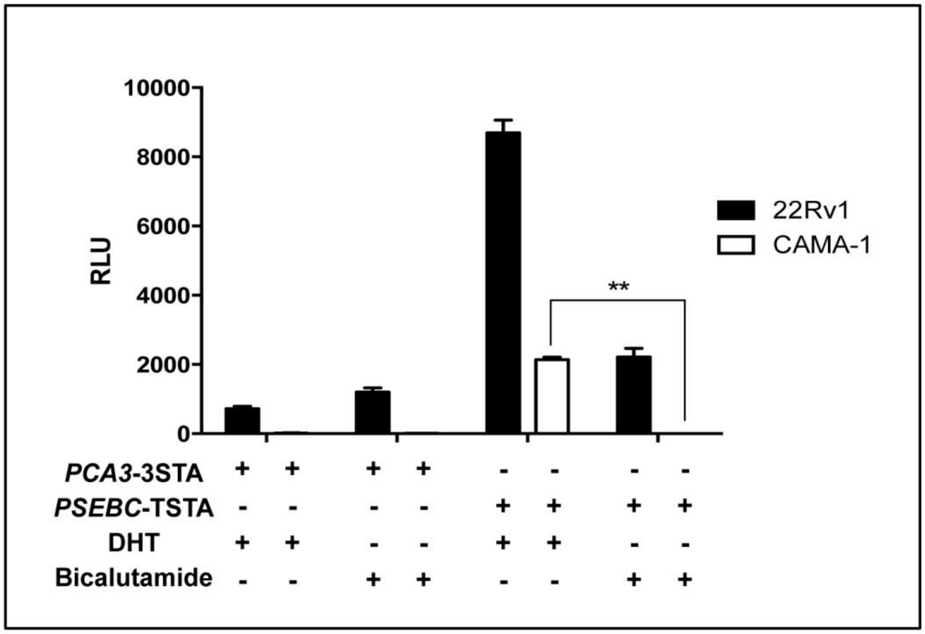

***Supplementary figure 2*: *PCA3*-3STA is prostate cancer specific and not induced by androgens but *PSEBC*-TSTA is highly induced by androgens.** Androgen receptor positive PCa cells (22Rv1) or androgen receptor positive breast cancer cells (CAMA-1) were infected with either *PSEBC*-TSTA or *PCA3*-3STA at 5 MOI and treated with either DHT (10 nM) or bicalutamide (10 μM). The relative fl activity (RLU) was normalized by protein content in each well divided by the SV40 promoter activity in each cell line (RLU = (RLU/μg protein) ÷ (RLU SV40/μg)*100).

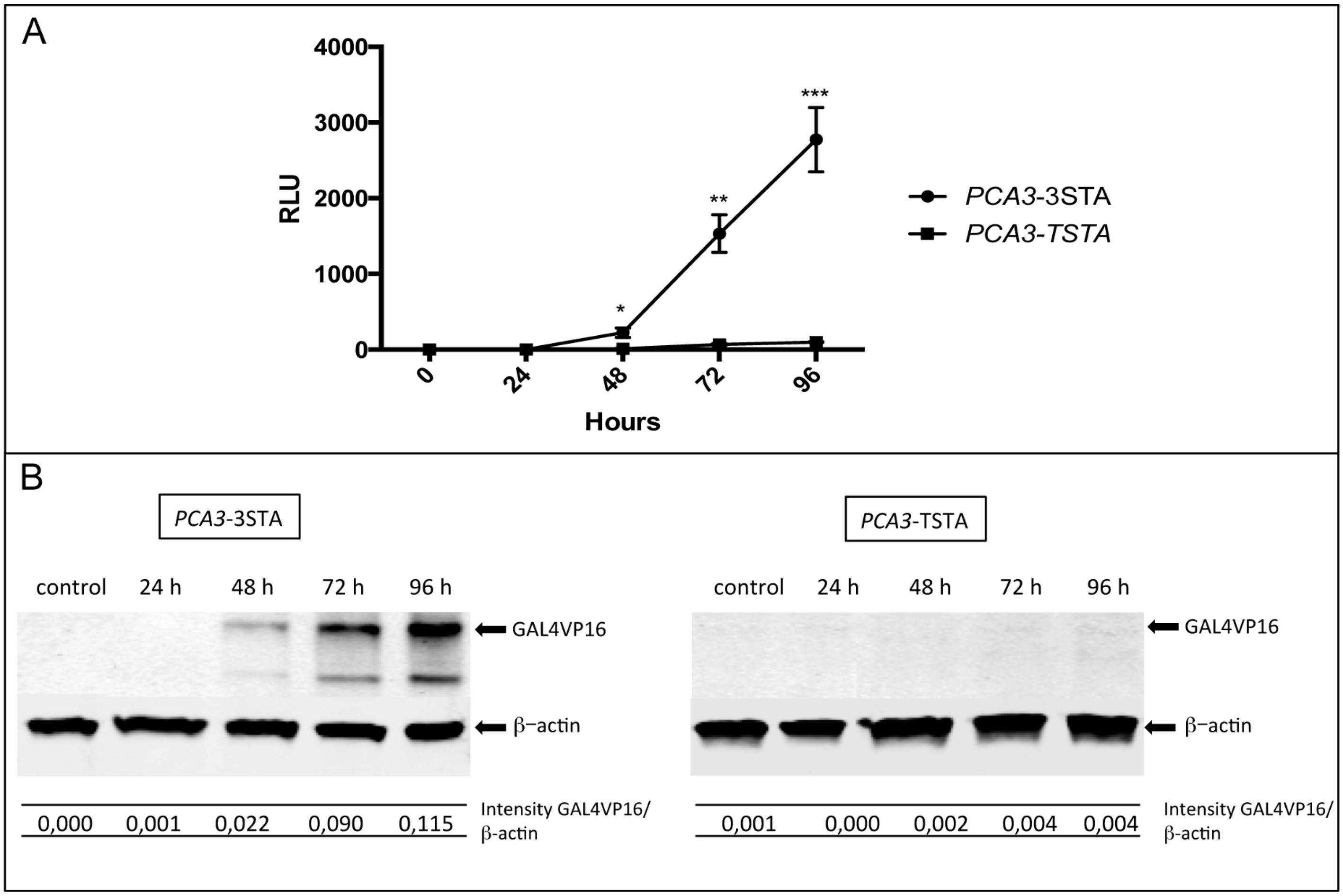

Supplementary figure 3: **The enhanced amplification provided by *PCA3*-3STA compared to that of TSTA was detected after 3 days and was secondary to increased GAL4VP16 protein expression.** **(A)** 22Rv1 cells were infected with the *PCA3*-TSTA and *PCA3*-3STA adenoviruses and were harvested at the indicated time points, and *fl* activity was measured. After 96 h, *PCA3*-3STA produced a higher signal than did *PCA3*-TSTA. The RLU was normalized by protein content in each well. Each data represents triplicates ± SD; *p < 0.05, **p < 0.01,***p < 0.001. **(B)** Cell extracts harvested above were analyzed by Western blot with anti-VP16 antibody. The intensity of the GAL4VP16 band was normalized for the β-actin levels.

Table S1

Clinical and histopathological informations of patients from which prostate tissue was sampled.

Patient number	Age (years)	PSA (ng/mL)	Surgery	Indication	Prostate cancer in sampled zone	Gleason score
**1**	65	8,2	Radical Prostatectomy	Prostate Cancer	Yes	3+4
**2**	70	8,4	Radical Prostatectomy	Prostate Cancer	Yes	4+3
**3**	58	5,9	Radical Prostatectomy	Prostate Cancer	Yes	3+4
**4**	76	10,0	Radical Prostatectomy	Prostate Cancer	Yes	4+3
**5**	74	–	Cystoprostatectomy	Bladder Cancer	No	–
**6**	68	–	Cystoprostatectomy	Bladder Cancer	No	–
**7**	73	–	Cystoprostatectomy	Bladder Cancer	No	–
**8**	79	–	Cystoprostatectomy	Bladder Cancer	No	–
**9**	83	–	Cystoprostatectomy	Bladder Cancer	No	–
